# Adipocyte-Cancer Cell Interactions in the Bone Microenvironment

**DOI:** 10.3389/fendo.2022.903925

**Published:** 2022-07-12

**Authors:** Meredith O. C. Otley, Christopher J. Sinal

**Affiliations:** Department of Pharmacology, Dalhousie University, Halifax, NS, Canada

**Keywords:** bone, adipocyte, cancer, adipokine, metastasis

## Abstract

When compared to adipocytes in other anatomical sites, the interaction of bone marrow resident adipocytes with the other cells in their microenvironment is less well understood. Bone marrow adipocytes originate from a resident, self-renewing population of multipotent bone marrow stromal cells which can also give rise to other lineages such as osteoblasts. The differentiation fate of these mesenchymal progenitors can be influenced to favour adipogenesis by several factors, including the administration of thiazolidinediones and increased age. Experimental data suggests that increases in bone marrow adipose tissue volume may make bone both more attractive to metastasis and conducive to cancer cell growth. Bone marrow adipocytes are known to secrete a variety of lipids, cytokines and bioactive signaling molecules known as adipokines, which have been implicated as mediators of the interaction between adipocytes and cancer cells. Recent studies have provided new insight into the impact of bone marrow adipose tissue volume expansion in regard to supporting and exacerbating the effects of bone metastasis from solid tumors, focusing on prostate, breast and lung cancer and blood cancers, focusing on multiple myeloma. In this mini-review, recent research developments pertaining to the role of factors which increase bone marrow adipose tissue volume, as well as the role of adipocyte secreted factors, in the progression of bone metastatic prostate and breast cancer are assessed. In particular, recent findings regarding the complex cross-talk between adipocytes and metastatic cells of both lung and prostate cancer are highlighted.

## Introduction

Cancer is a leading cause of death worldwide that is attributable for an estimated 14 million incident cases and 8 million deaths annually (1, 2). While obesity is now recognized as a risk factor for several malignancies (3, 4), pathophysiological context and other biological factors modify the magnitude of this effect. Obesity is characterized not only by adipose tissue expansion, but also a progressive adipose tissue dysfunction resulting in profound alterations in the production of lipids, hormones, inflammatory cytokines and adipose derived-signalling molecules termed adipokines (5). These local and systemic physiologic alterations have the potential to impact cancer cells indirectly through immunomodulation or modulation of the tumour microenvironment as well as directly *via* effects on cancer cell growth (6). While considerable study has been devoted to the influence of adipocytes localized in white adipose tissue (WAT) depots (e.g. subcutaneous) on cancer development and progression, comparatively little attention has been paid to adipocytes resident within bone marrow. This is important, as these adipocytes have unique metabolic and paracrine/endocrine features as well as a developmental origin distinct from peripheral white adipocytes. Moreover, given their location within bone marrow, these adipocytes may be particularly relevant to promoting bone metastases and/or supporting the growth of metastatic cancer cells. This minireview highlights recent findings regarding the role of bone marrow adipocytes in the skeletal metastasis.

## Origin and Physiological/Pathological Relevance of Bone Marrow Adipocytes

The presence of fat within bone marrow has long been recognized through gross anatomical and histological findings (7). At birth, bone marrow is largely red in appearance owing to the preponderance of hematopoietic and osteogenic cells (8). Beginning in childhood, there is a gradual yellowing of the bone marrow, first in the long bones, and eventually other skeletal sites, due to an accumulation of adipocytes within the marrow (8). While there is considerable individual variation, at the population level there is in general a positive correlation between the volume of bone marrow adipose tissue (BMAT) and age (9). In particular, females experience a marked increase of BMAT between the ages of 55-65 (10). Epidemiological evidence supports a linkage between this increase in BMAT and age-related bone loss (e.g. osteoporosis), particularly in post-menopausal women (11, 12). In addition to age, commonly used drugs such as glucocorticoids and thiazolidinediones have been linked to increased BMAT and bone loss (13). Data regarding the relationship between BMAT and total body fat or specific WAT depots (e.g. visceral, subcutaneous) in humans is somewhat inconsistent with some studies reporting a positive, and others no association with amounts of BMAT (14, 15). Adding to the complexity of this relationship, other studies have reported that conditions characterized by decreased WAT, such as anorexia nervosa and caloric restriction in rodents, are associated with increase BMAT volume (16–18).

It is generally accepted that bone marrow adipocytes derive from a self-renewing population of multipotent progenitor bone marrow stromal cells (BMSCs) (19). Most evidence supports that this developmental origin is distinct from other adipocytes (e.g. subcutaneous white adipocytes) (20, 21) and contributes to the unique role of bone marrow adipocytes in local processes such as hematopoiesis (22) and osteogenesis (23) as well as energy metabolism at both the local and systemic level (24). In addition to adipocytes, BMSCs can also give rise to other bone cell types including chondrocytes, myocytes, and osteoblasts (19) depending upon the nature of the paracrine/endocrine stimulus. Indeed, a shift in BMSC lineage allocation to favour adipogenic versus osteogenic differentiation is believed to contribute to aging and post-menopausal bone loss (25). Similar to white adipocytes, bone marrow adipocytes secrete a variety of biologically active signalling molecules including pro-inflammatory cytokines and adipokines with local and systemic effects. For example, bone marrow adipocytes secrete tumour necrosis factor-alpha and adiponectin, both of which have been shown to inhibit bone marrow hematopoiesis (26). Also, in common with WAT, BMAT serves as an energy reservoir, largely in the form of triglycerides, that can be mobilized through lipolysis and released as free fatty acids (FFAs) into the extracellular environment. While these FFAs may serve as a general energy source that is supportive of normal physiological processes such as hematopoiesis and bone remodelling (24), they may also have unique properties and pathological relevance to the bone marrow microenvironment. For example, elevated levels of certain bone marrow adipocyte-derived saturated FFAs, such as lauric and palmitic acid, inhibit osteoblastic differentiation of BMSCs, promote osteoclast survival and in doing so, may contribute to the potential linkage between BMAT and bone loss (27). Emerging evidence indicates that the pathological relevance of BMAT may also extend to several malignancies and that an adipocyte-rich bone marrow may be both attractive and supportive of metastatic cancer cells (28). This review will focus on prostate, breast and lung cancer and multiple myeloma as the interaction between BMAT and the metastasis of these malignancies has been the most widely studied and thus, most amenable for summation and analysis.

## Prostate Cancer

**Figure 1:**
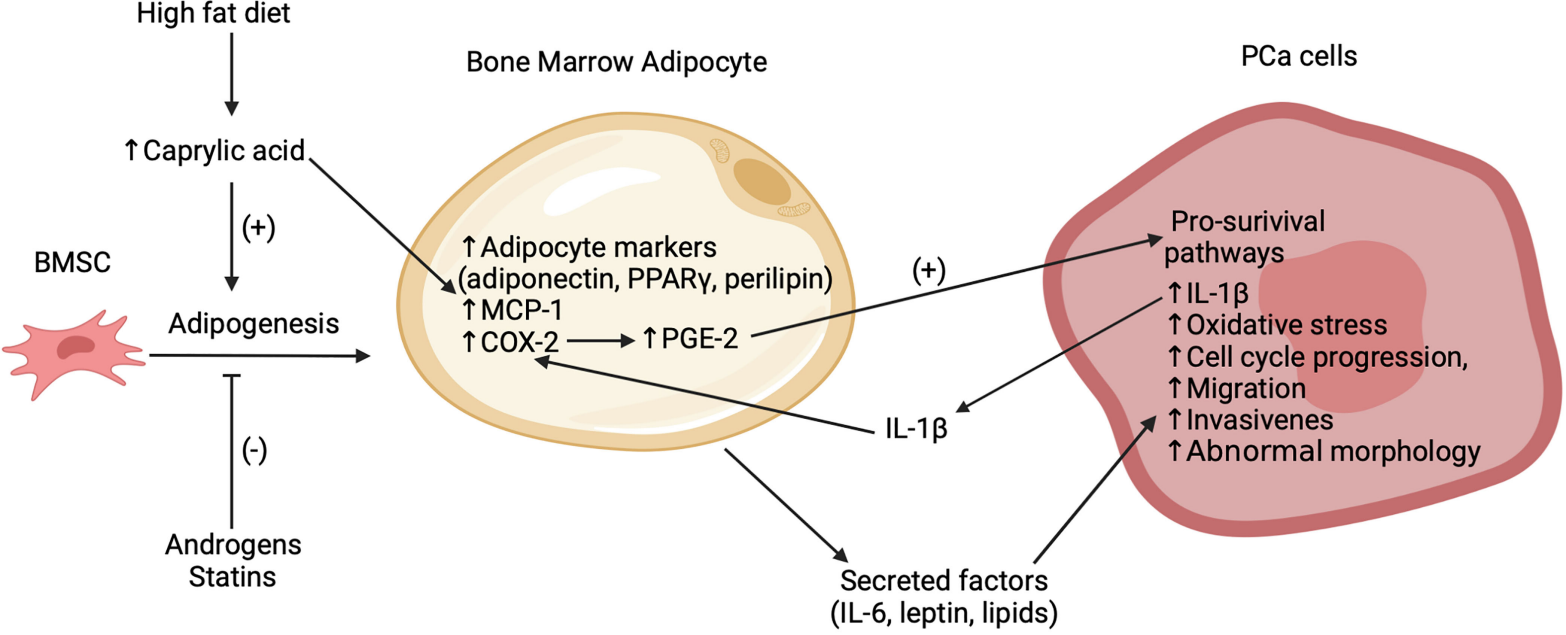
Summary of recent findings regarding the interactions between bone marrow adipocytes and PCa cells in the bone marrow microenvironment. Bone marrow adipogenesis promoted by high fat diet, caprylic acid and inhibited by statins and androgens. Caprylic acid increases adipocyte markers (adiponectin, PPARγ, perilipin), MCP-1 and COX-2 expression in the bone marrow adipocytes. COX-2 subsequently increases PGE-2 which activates pro-survival pathways in the bone metastatic PCa cells. Secreted factors, which potentially include leptin and lipids, stimulate oxidative stress, cell cycle progression, migration, invasion, abnormal morphology, and secretion of IL-1β in the PCa cells. This IL-1β acts to further increase COX-2. While this figure highlights empirical findings from PCa studies, it is likely that several of these mechanisms are also relevant to the interaction of bone marrow adipocytes with other cancer cell types.

Bone is the most common metastatic site in prostate cancer (PCa) (29). Several studies have implicated bone marrow adipocytes as key facilitators of the progression and exacerbation of these bone metastases, as summarized in **Figure 1** (30–34). Two recent preclinical trials have emphasized the association between an increased number bone marrow adipocytes, volume of BMAT and the progression of PCa cells in the bone marrow niche (32, 33). High fat diet, caprylic acid treatment and androgen depletion (castration) enhanced the BMSC-to-adipocyte transition *in vitro* and in mouse models (32, 33). Androgen deprivation therapy (ADT) is a common treatment for prostate cancer. Clinical studies have demonstrated an increase in BMAT and decrease of bone mineral density in lower spinal vertebral bodies (35) as well as an increased risk for the development of castrate-resistant bone metastases for patients undergoing this treatment (36). Pan et al. (32) found that the bone marrow of castrated mice compared to controls had increased bone marrow adipocytes as well as increased levels of adipocyte markers, most notably adiponectin, perilipin and peroxisome proliferator-activated receptor-γ (PPARγ). Moreover, treatment of BMSCs *in vitro* with androgens suppressed adipogenesis and transient knockdown of the androgen receptor inhibited this suppression (32). These findings are consistent with previous clinical findings of increased marrow fat fraction after ADT (35, 37). Interestingly, treatment with statins, inhibitors of cholesterol biosynthesis, suppressed adipogenesis *in vitro* and *in vivo* and reduced bone metastatic PCa progression in a 22RV1/LT xenograft model with castrated mice (32). These effects were found to be mediated in part through a reduced expression of both PPARγ - a pro-adipogenic transcription factor – and leptin – an adipokine that was shown to promote cell-cycle progression and proliferation of PCa cells – after statin treatment. These data suggest the potential therapeutic utility of agents that interrupt the BMSC-to-adipocyte transition as a novel approach to reduce PCa metastasis.

Previous studies have suggested that high fat diets can promote adipogenesis in bone and thereby contribute to a pro-tumor environment (31, 38). However, the mechanisms underlying this effect remain unclear. Wang et al. (33) found that mice on a high fat diet exhibited increased marrow adiposity and FFA levels. Following from this, they identified caprylic acid as a specific FFA with levels higher in the blood of patients with PCa bone metastasis *versus* that of PCa patients without bone metastasis or that of healthy controls. However, it should be noted that these data are derived from a relatively small sample size with 16 controls, 8 patients with PCa and 8 patients with PCa bone metastasis. While it is unclear as to whether the relationship of the elevated caprylic acid levels in patients was causative or correlational with PCa bone metastasis, *in vivo* treatment of BMSCs with caprylic acid increased the adipocyte differentiation and the protein expression of PPARγ, while subsequently reducing the number of osteoblasts. Treatment of bone marrow adipocytes with caprylic acid also induced higher expression of cyclooxygenase 2 (COX-2) (33), which has been implicated in several pro-tumor pathways (39). Thus, elevated levels of caprylic acid may support PCa bone metastasis through the promotion of adipogenesis over osteoblastogenesis. Similar results have been reported for arachidonic acid (40), suggesting that elevated FFA levels due to high fat diets may contribute to PCa bone metastasis by promoting a pro-tumor environment *via* increased bone marrow adipocytes and BMAT volume.

It is well established that certain factors secreted by white adipocytes, such as interleukin-6 (IL-6), contribute to primary PCa progression (41). Less well defined is the contribution of adipocyte secreted factors to the pro-tumor microenvironment within bone marrow. Both bone marrow adipocyte-derived lipids (38) and certain adipokines (42) have been shown to have several pro-tumor actions with respect to PCa cells including stimulating cell cycle progression, proliferation (32), migration, invasion (30, 33), abnormal morphology (43) and promoting oxidative stress (30). For example, in a 3D *in vitro* co-culture model of bone marrow adipocytes and PCa cells, inhibitors of fatty acid-binding protein 4 and adipocyte triglyceride lipase, used in combination, reduced the invasiveness of PCa cells (43). While it is unclear if the effect of fatty acid-binding protein 4 inhibition was a consequence of effects in the adipocytes and/or PCA cells, given the high levels of adipocyte triglyceride lipase expression in adipocytes, it is likely that reduced PCa invasiveness was indirectly liked to inhibition of adipocyte lipolysis. Overall, these data are consistent with previous findings from a 2D co-culture model that lipolysis and subsequent uptake of bone marrow adipocyte-derived lipids by PCa cells may be a key mediator of the interaction between these cell types (38). The adipokine leptin was found to be increased in bone marrow adipocyte-conditioned media and treatment with recombinant leptin stimulated PCa cell cycle progression and proliferation, possibly mediated by activation of STAT3, a transcription factor that has been implicated in promoting the survival, growth and metastasis of cancer cells (32, 44). While these initial findings are informative, the identities and mechanisms of action of secreted factors that mediate the interactions between bone marrow adipocytes and PCa cells require further investigation.

Although increased levels of reactive oxygen species are generally detrimental to cells, persistent activation of oxidative stress pathways by bone marrow adipocytes, may promote tumor progression in a tissue-selective manner (30, 45). For example, heme oxygenase 1 (HO-1) was reported to be upregulated in PCa bone tumors but not in subcutaneous tumors of mice with diet induced marrow adiposity, indicating an effect specific to the bone marrow environment (30). Upon review of ONCOMINE, a cancer microarray database, Herroon et al. subsequently reported (30) that HO-1 is elevated in metastatic human PCa tumors compared to primary tumors. However, they were not able to distinguish between bone metastases and other metastatic sites using that database. Based on investigation of metastatic PCa cells in co-culture with bone marrow adipocytes, HO-1 overexpression was linked to pro-survival pathways in PCa cells, an effect which was significantly reduced by treatment with an HO-1 inhibitor (30). Moreover, consistent with other findings (38), bone marrow adipocytes increased the invasive potential of PCa cells. This effect was reduced by antioxidant treatment and subsequently recovered by forced overexpression of HO-1 in PCa cells (30). While these data point to antioxidants as a potential strategy for treating bone metastasis in PCa, a recent systematic review indicated that the efficacy of antioxidant supplements taken by cancer patients remains unclear (46). In addition to oxidative stress, two recent studies from Heroon et al. (30, 34) have examined the upregulation of markers of endoplasmic reticulum (ER) stress in PCa cells upon interaction with bone marrow adipocytes. Their findings suggest that ER chaperone glucose regulated protein 78 (BIP), may be involved in facilitating bone marrow adipocyte-mediated ER stress in metastatic PCa cells which may help them survive in the bone marrow environment.

Emerging evidence indicates that the interaction between PCa cells and bone marrow adipocytes is not one sided, but rather entails a complex cross talk involving multiple paracrine factors. Interleukin 1β (IL-1β) was reported to be upregulated in bone metastatic PCa cells in mice with diet induced bone marrow adiposity (38). Another study found that this cytokine upregulated the expression of COX-2, as was also observed with caprylic acid treatment (33), and macrophage chemoattractant protein (MCP-1) in bone marrow adipocytes (31). This COX-2 upregulation was further linked to an increase in the production of prostaglandin-2 (PGE-2) by bone marrow adipocytes which was proposed to reciprocally act on PCa to activate pro-survival pathways (31, 33). Interestingly, PCa cells co-cultured with bone marrow adipocytes exhibited a reduced sensitivity to docetaxel, a drug used to treat metastatic PCa (31). The response to docetaxel was partially restored by inhibition of IL-1β or lipolysis, suggesting that BMA-PCa cross talk may be linked to drug resistance. Further investigation is needed in order to determine the implications of these findings.

## Breast Cancer

Similar to PCa, bone is the most common metastatic site for breast cancer (47). The primary site of breast cancer growth is in close vicinity to the mammary fat pad and there is a well-established link between these local white adipocytes and breast cancer development (48). It has been proposed that this connection also holds true with respect to the interaction between bone marrow adipocytes and bone metastatic breast cancer cells (48). However, this notion is challenged somewhat by known phenotypic distinctions between white adipocytes and bone marrow adipocytes (49), as well as the limited number of bone marrow adipocyte-specific studies addressing this relationship. Recent data has demonstrated that there is preferential migration of breast cancer cells towards bone marrow adipocytes occupied components when compared to the mineralized component of bone in a human bone tissue explant model (50). This behaviour was exhibited by two different breast cancer cell lines (MCF-7 and MDA-MB-231) and there was extensive direct contact between the breast cancer cells and the bone marrow adipocytes (50). MDA-MB-231 cells, a bone-trophic line, were found to overexpress HO-1 after co-culture with bone marrow adipocytes (30). Similarly, increased proliferation and invasion of MDA-MB-231 cells after exposure to bone marrow adipocyte-conditioned media was also associated with HO-1 induction in a previous study (38).While there is evidence that HO-1 upregulation led to the activation of pro-survival pathways in PCa cells, a similar mechanism remains to be demonstrated in breast cancer cells. In an analysis of 56 breast cancer patients and 56 controls, high BMAT volume was found to be an independent risk factor for breast cancer (51). However, while it was strongly associated with lymph node metastasis, the study did not include bone metastasis as a parameter (51).

While bone marrow adipocyte-secreted factors have been suggested as mediators of the interaction between this cell type and breast cancer cells, empirical evidence to support this is currently limited. Cytoplasmic lipid accumulation and upregulation of lipid transport proteins was reported for MDA-MB-231 cells exposed to either bone marrow adipocyte-conditioned media or in transwell co-culture with bone marrow adipocytes (38). Increased uptake of bone marrow adipocyte-derived FFAs by *via* fatty acid binding protein-4 was proposed as a means of energy provision to support the increased proliferation and invasiveness of breast cancer cells observed under these conditions (38). However, there have been no further studies supporting this hypothesis. Breast cancer cells have been observed to exhibit increased migration toward the bone marrow adipocytes in an explant model in which MCF-7 or MDA-MB-231 cells were co-cultured with cancellous bone tissue fragments isolated from hip arthroplasties (50). Analysis of the supernatants of the explants revealed a significant association between increasing levels of IL-1β and leptin with MDA-MB-231 migration in the bone microenvironment and implicate these factors as potential mediators by which bone marrow adipocytes encourage breast cancer cell migration to bone marrow (50). However, there are some limitations to this conclusion, as it was assumed that bone marrow adipocytes were the main source of leptin and IL-1β in the bone marrow environment. These findings, support that increased BMAT volume in post-menopausal women (10) may contribute to breast cancer bone metastasis and other clinical findings indicative of a poorer prognosis for post- versus pre-menopausal breast cancer patients (52). However, other clinical data suggests that there is a lower incidence of bone metastasis in post- versus pre-menopausal women (53). As such, this relationship remains unclear at present and further clinical investigation of the impact of increased post-menopausal marrow adiposity on bone metastasis of breast cancer is needed.

## Lung Cancer

As bone metastasis is less common in lung cancer than in breast and prostate cancer, there has been little investigation regarding the role of bone marrow adipocytes in the progression of lung cancer (47). However, a recent study investigated the cross talk between bone marrow adipocytes and small cell lung carcinoma cells (54). Rosiglitazone maleate, a thiazolidinedione, was used to induce increased BMAT volume in mice. Compared to controls that did not receive rosiglitazone treatment, the treated mice exhibited augmented osteolytic destruction when SBC-5 cells, a small cell lung carcinoma cell line with bone metastatic potential, were injected into their femurs (54). S100A8/A9 is a heterodimeric calcium binding protein that is highly expressed in several cancer types and is involved in the regulation inflammatory processes and immune response (54, 55). In comparing SBC-5 to SBC-3 (a small cell lung carcinoma cell line without bone metastatic potential), increased S100A8/A9 expression was the predominant difference identified in the SBC-5 versus -3 transcriptome (54). These data suggest that S100A9/A8 expression levels may be a determinant of the bone tropism of certain lung cancers. Moreover, co-culture of bone marrow adipocytes and SBC-5 cells, was associated with elevated expression of IL-6 in the former and the cognate receptor (IL-6R) in the latter. Conditioned media from BMSCs enhanced the migration of both SBC-3 and SBC-5 cells, whereas conditioned media from bone marrow adipocytes enhanced the invasion of SBC-5 cells only. Reciprocally, SBC-5 conditioned media inhibited the adipogenic differentiation of BMSCs and promoted de-differentiation and decreased adipogenic marker expression in mature bone marrow adipocytes. This effect was reduced when toll-like receptor 4, which can be activated by S100A8/A9 (56), was inhibited, further implicating S100A8/A9 in this cross talk. More research is required to better understand these findings and their implications for the progression and maintenance of bone metastatic lung cancer.

## Multiple Myeloma

Oncolytic bone loss is a frequent occurrence with multiple myeloma and while several comprehensive reviews (57–59) have addressed and summarized findings regarding the complex cross-talk between marrow adipocytes and myeloma cells, recent studies further characterizing this relationship are worth highlighting. For example, while it has been variously reported that multiple myeloma cells can promote the adipogenic versus osteoblastogenic differentiation of MSCs, the mechanisms underlying this effect have been unclear. Liu et al. (60, 61) recently described a mechanism by which the integrin alpha4 subunit expressed on the surface of multipole myeloma cells stimulated vascular cell adhesion molecule 1 on MSCs leading to repression of muscle ring-finger protein-1 mediated ubiquitination of PPARγ. The resultant stabilization and accumulation of PPARγ levels in turn promoted adipogenesis and reduced osteoblastogenesis of MSCs, suppressing bone formation *in vitro* and *in vivo*. Another recent study (60) reported that conditioned media prepared from adipocytes isolated from bone marrow aspirates collected from myeloma patients (newly diagnosed or in complete remission) promoted the development of prominent osteolytic lesions in a humanized murine fetal bone chip model when compared to media prepared from adipocytes of normal subjects. Interestingly, co-culture of multiple myeloma cells with MSC-derived adipocytes resulted in the development of a “senescence-associated secretory” phenotype characterized by alterations in the release of adipose-derived cytokines, adipokines and other signalling molecules associated the promotion and survival of tumour cells (62). Consistent with a tumour supportive relationship between BMAT and multiple myeloma, clinical studies have found that inclusion of bone marrow fat fraction improved both the discrimination of healthy controls from multiple myeloma patients by MRI and further, those patients with diffuse versus focal lesions (63). Taken together, these findings are consistent with a complex cross-talk between multiple myeloma cells and adipocytes. This may entail a reprogramming of MSCs and adipocytes, that persists even with remission, to repress bone formation and promote oncolytic lesions as well as to support tumour growth and survival.

## Discussion

The prognosis for cancer patients with bone metastasis remains poor. Currently, most research investigating the interactions between bone marrow adipocytes and bone metastasis has focused on prostate cancer and multiple myeloma. However, given the high incidence of bone metastasis in breast cancer patients, as well as the clear relationship between white adipocytes and primary breast tumors, this is an important area of future study. Despite the lower incidence of bone metastasis in lung cancer compared to that seen in breast and prostate cancer, it has a shorter median survival (47), illustrating the need for a greater understanding of the role of bone marrow adipocytes in lung cancer bone-tropism. In addition to further research into specific cancer types, identification of which secreted factors mediate these interactions and the elucidation of the underlying mechanisms is needed. Based on the current research, there could be potential therapeutic implications if strategies to reduce the BMSC-to-adipocyte transition or to interrupt adipocyte-tumor cross talk are more clearly defined.

## Author Contributions

MO and CS contributed equally to the conception and preparation of this manuscript.

## Funding

The authors’ work was supported by funding from Research Nova Scotia.

## Conflict of Interest

The authors declare that the research was conducted in the absence of any commercial or financial relationships that could be construed as a potential conflict of interest.

## Publisher’s Note

All claims expressed in this article are solely those of the authors and do not necessarily represent those of their affiliated organizations, or those of the publisher, the editors and the reviewers. Any product that may be evaluated in this article, or claim that may be made by its manufacturer, is not guaranteed or endorsed by the publisher.
